# Analysis of risk factors for deep venous thrombosis in patients with gynecological malignant tumor: A clinical study

**DOI:** 10.12669/pjms.35.1.365

**Published:** 2019

**Authors:** Qun Li, Yinling Xue, Yuan Peng, Lin Li

**Affiliations:** 1 Qun Li, Department of Acupuncture and Rehabilitation, Qingdao Hiser Medical Group, Qingdao, Shandong Province, 266033, China; 2 Yinling Xue, Department of Acupuncture and Rehabilitation, Qingdao Hiser Medical Group, Qingdao, Shandong Province, 266033, China; 3 Yuan Peng, Department of Acupuncture and Rehabilitation, Qingdao Hiser Medical Group, Qingdao, Shandong Province, 266033, China; 4 Lin Li, Department of Vascular Surgery, Qingdao Hiser Medical Group, Qingdao, Shandong Province, 266033, China

**Keywords:** Deep venous thrombosis (DVT), Gynecological malignant tumor, Risk factors

## Abstract

**Objectives:**

To determine the clinical characteristics and risk factors of Deep Venous Thrombosis (DVT) in patients with gynecological malignant tumor, facilitating gynecologists better prevent the fatal complication.

**Methods:**

The patients with gynecological malignant tumor treated in department of gynecology of our hospital between May 2013 and May 2018 were reviewed retrospectively. The clinical data of patients including gender, age, tumor staging, adenocarcinoma, surgery, operation time, hypertension, hyperlipemia, diabetes, coronary heart disease, radiotherapy, chemotherapy, hospital stay, and postoperative rehabilitation exercise were collected to analyze the clinical characteristics of patients and determine the risk factors of DVT.

**Results:**

In the current study, 67 patients were included in DVT group, and 554 patients were included in Non-DVT group. There were significant differences in age, hypertension, hyperlipemia, operation time, adenocarcinoma, tumor staging, radiotherapy and postoperative rehabilitation exercises between DVT and non-DVT groups (p<0.05). However, there was no significant differences in gender, coronary heart disease, diabetes, surgical treatment and hospital stay (p>0.05). In multivariate analysis, the factors including age, hypertension, adenocarcinoma, radiotherapy, and hyperlipemia were independent risk factors, while rehabilitation exercise was protective factor for DVT.

**Conclusion:**

In cases of gynecological malignant tumor, DVT screening should be given due importance, especially for those patients with old age, hypertension, hyperlipemia, adenocarcinoma, or history of radiotherapy. Rehabilitation exercise should be encouraged in these patients.

## INTRODUCTION

Gynecological cancer is the leading cause of cancer-related death in women^1^, and deep vein thrombosis (DVT) is one of the most common complications in patients with gynecological cancer. In a study of 87 patients with ovarian cancer, Kawaguchi reported the incidence of DVT was 16.1%.^2^ More profoundly, some patients with DVT may develop into pulmonary embolism (PE), which leads to a high mortality rate.^3^ Gynecological malignant tumor usually needs surgical treatment, which may aggravate the occurrence of DVT. Geerts reported the risk of DVT after gynecologic surgery was high up to 17-40%.^4^ Subsequently, the fatal complication is given due importance in gynecological department.^5^

Clinically, some precautionary measures have been performed to prevent the occurrence of DVT, but it still occurs frequently. Also, the clinical diagnosis of this disease is not so accurate.^5^ Subsequently, it is critical to study the clinical characteristics and risk factors for the fatal disease. Some authors have carried out lots of studies in this field. In a study of four hundred and ninety-eight patients with gynecological malignant tumor treated surgically, Zhang and colleagues concluded that age, cardiovascular comorbidity and postoperative hemostatics dose were independent risk factors for DVT.^5^ However, some authors held different viewpoints, in another study of 120 cases with gynecological malignant tumor treated surgically or conservatively, Yan suggested that hyperlipidemia, diabetes, pathological type of cancer, surgery and radiotherapy were the independent risk factors of DVT in patients with gynecological malignant tumor.^6^ In addition, Huang^7^ also focused on the similar study, but his conclusion was different from the two above-mentioned studies. The current conditions may affect gynecologists understand the fatal complication adversely, and it need more studies to clarify the issue.

Therefore, we carried out a retrospective study in patients with gynecological malignant tumor treated surgically or non-surgically between May 2013 and May 2018. The aim of this study was to determine the clinical characteristics and risk factors of DVT in patients with gynecological malignant tumor, facilitating gynecologists better prevent the fatal complication.

## METHODS

In this study, the patients with gynecological malignant tumor treated in department of gynecology of our hospital between May 2013 and May 2018 were reviewed retrospectively. The inclusion criteria were: 1) patients who were diagnosed with gynecological malignant tumor such as cervical cancer, endometrial cancer, ovarian cancer, and fallopian tube cancer, treated surgically or non-surgically, 2) patients who had no history of DVT, and 3) patients with complete clinical data. DVT was diagnosed based on imaging examinations, including Doppler ultrasound, magnetic resonance imaging venography or angiography, as well as plasma D-dimer.^8^ The patients who had DVT history, or who developed arterial thrombosis, or with a history of blood disease, or those without complete clinical data, were excluded from the study. The study was approved by the Ethics Committee of our hospital.

The clinical data of patients including gender, age, tumor staging, adenocarcinoma, surgery, operation time, hypertension, hyperlipemia, diabetes, coronary heart disease, radiotherapy, chemotherapy, hospital stay, and postoperative rehabilitation exercises were collected to analyze the clinical characteristics of patients and determine the risk factors of DVT. In these clinical data, hypertension was defined as blood pressure above 140/90 mmHg^9^, and hyperlipemia was defined as an elevated total cholesterol level above 6.22 mmol/L, or triaglyceride level above 2.26 mmol/L, or low-density lipoprotein level above 4.14 mmol/L^10^, and other disease such as diabetes and coronary heart disease were also diagnosed according to corresponding criteria.^11^,^12^ Most of the included patients in the current study were treated surgically, to facilitate to determine the influence of surgical treatment on the occurrence of DVT, the thrombosis occurring before or after surgery was identified clearly.

The statistical analysis was carried out using SPSS 21.0 (SPSS Inc., Chicago, IL, United States). The assessment of categorical variables was performed using chi-squared test, measurement data was compared with analysis of variance, and univariate and multivariate logistic regression analysis were performed to detect the correlation between variables and DVT. Multivariate logistic regression analysis was used to determine the independent risk factors for DVT. A *P* value < 0.05 was regarded as statistical significance.

## RESULTS

In the current study, 698 patients with gynecological malignant tumor were treated in the department of gynecology. Most patients received surgical treatment. Seventy seven patients were excluded for failing to meet the inclusion criteria, and 621 were included in this study. Among the included patients, 577 were treated with radical surgery, and 44 were treated using conservative treatment because they had advanced tumor stage and were not amenable to surgical resection. Sixty seven patients were diagnosed with DVT and included in DVT group, the remaining 554 patients without DVT were included in Non-DVT group. The incidence of DVT was 10.8% in this study. The clinical characteristics of the patients in two groups are shown in Table-I.

**Table-I:** Clinical characteristics of the two groups.

Factors	DVT	Non-DVT	P value

	n=67	n=554	
Age (> 55 years) (n, %)	55(82.1%)	285(51.4%)	0.0002
Gender (M/F)	35/32	310/244	0.56
Hypertension (n, %)	47(70.1%)	261(47.1%)	0.0003
Hyperlipemia	45(67.2%)	284(51.3%)	0.01
Coronary heart disease (n, %)	36(53.7%)	257(46.4%)	0.26
Diabetes (n, %)	11(16.4%)	121(21.8%)	0.31
Adenocarcinoma (n, %)	41(61.2%)	267(48.2%)	0.04
Tumor staging			0.00
Stage I	5	152	
Stage II	11	218	
Stage III	23	137	
Stage IV	28	47	
Surgical treatment (n, %)	65(97.0%)	512(92.4%)	0.16
Operation time>3 hours (n, %)	38(56.7%)	389(70.2%)	0.02
Rehabilitation exercises (n, %)	19(28.4%)	251(45.3%)	0.008
Chemotherapy (n, %)	34(50.7%)	260(46.9%)	0.55
Radiotherapy (n, %)	37(55.2%)	219(39.5%)	0.01
Hospital stay(days)	28.9±5.2	22.7±6.2	0.07

There were significant differences in age, hypertension, hyperlipemia, operation time, adenocarcinoma, tumor staging, radiotherapy and postoperative rehabilitation exercises between DVT and non-DVT groups (p<0.05) but no significant differences in gender, coronary heart disease, diabetes, surgical treatment and hospital stay (p>0.05, Table-I).

In multivariate analysis, the factors including age, hypertension, adenocarcinoma, radiotherapy, and hyperlipemia were independent risk factors, while rehabilitation exercises were protective factor for DVT (Table-II).

**Table-II:** The independent risk factors for deep venous thrombosis.

Factors	P value	OR (95% CI)
Age (> 55 years)	0.000	4.326(2.267-8.256)
Hypertension	0.000	2.638(1.523-4.539)
Hyperlipemia	0.014	1.945(1.009-2.848)
Adenocarcinoma	0.03	1.695(0.201-0.743)
Rehabilitation exercises	0.005	0.478(0.274-0.834)
Radiotherapy	0.010	1.887(1.132-3.144)

## DISCUSSION

In the current study, the clinical data of 621 patients with gynecological malignant tumor in department of gynecology of our hospital were analyzed to determine the risk factors of DVT. This study may help gynecologists understand the occurrence of the fatal disease and make correct treatment strategies.

In our multivariate analysis, the factors including age, hypertension, hyperlipemia, radiotherapy, and adenocarcinoma were independent risk factors for DVT in patients with gynecological malignant tumor. In a review of 9146 patients undergoing anterior cruciate ligament reconstruction, Bokshan suggested that increasing age over 30 years and hypertension were associated with an increased risk of DVT.^13^ Alanazi’s study also drew the same conclusion.^14^ Elderly population are usually associated with vascular sclerosis, high blood viscosity and poor venous valve function, this may be the reason for a high rate of DVT.^8^ In terms of hypertension, its association with the development of DVT is a controversial issue. Some previous studies suggested that hypertension could increase the risk of DVT^6^, but some other studies concluded that there was no statistically significant association between it and DVT.^15^ However, in the current study, we confirmed that hypertension was an independent risk factor for DVT in patients with gynecological malignant tumor.

As regards hyperlipidemia, in a study of 59 consecutive DVT patients, Kawasaki found the incidence of hyperlipidemia was high, as compared with normal control, suggesting that hyperlipidemia was an important etiologic factor in DVT.^16^ In this study, 67.2% of patients in DVT group suffered from hyperlipemia, but the percentage in non-DVT group was only 51.3%, a significant difference was found between two groups and the multivariate analysis confirmed it was an independent risk factor for DVT.

Meanwhile, radiotherapy was also found to be an independent risk factor for DVT in this study, but some authors held different viewpoints. In a study of 272 consecutive patients with cervical cancer, Satoh concluded that radiotherapy can reduce the occurrence of venous thromboembolism.^17^ In our opinion, Satoh’s study had a relatively small sample and relatively low rate of DVT, which may affect the final outcomes. At the same time, we found that tumor type may significantly affect the occurrence of DVT and adenocarcinoma was a risk factor for the complication. Cancer is associated with a high incidence of DVT,^18^ among which adenocarcinoma with mucinous component has an even higher propensity to cause thrombosis, this conclusion has been confirmed by many physicians.^19^

Moreover, some researchers suggested that surgical treatment was one of the independent risk factors for DVT. In a study of four hundred and ninety-eight patients with gynecological disease treated surgically, Zhang and colleague found the incidence of deep venous thrombosis was up to 11.6%, demonstrating a high incidence of DVT in patients after gynecological surgery.^5^ They suggested that anaesthesia during surgery may cause venous distension, postoperative long bed rest period may affect the hemodynamics, and the trauma resulted from surgery may aggravate the blood hyper coagulation negatively.^5^ Subsequently gynecological surgery lead to a high incidence of DVT. Yan also held the same viewpoint.^6^ In the current study, among 621 included patients, 577 patients received tumorectomy, and only 44 patients were treated conservatively. The rate of surgical treatment in DVT and non-DVT group was 97% and 92.4%, respectively. Although the percentage of surgical treatment in DVT group was higher, no significant difference was detected between the two groups. We believe, this is attributed to the small number of patients treated conservatively in the current study.

In addition, we found rehabilitation exercise was a protective factor for DVT, which played an important role in the prophylaxis of the disease. The incidence of DVT in patients with rehabilitation exercises was significantly lower as compared to others. Some published literature also supports the viewpoint. In a retrospective study of consecutive patients undergoing total hip arthroplasty, 126 patients underwent manual calf massage and passive ankle motion, and 138 patients underwent total hip arthroplasty using the same surgical approach without this mechanical prophylaxis, Imai and colleagues found the incidence of deep vein thrombosis was 6.52% and 0.79% in the control and rehabilitation groups, respectively, indicating performing this mechanical prophylaxis reduced the incidence of venous thromboembolism after total hip arthroplasty.^20^ Many reports showed an increase in femoral vein blood flow during active ankle exercises^21^, which lead to a reduced rate of DVT in the rehabilitation group. In the current study, although the included patients were different, but the mechanism of rehabilitation exercises for prevention of DVT are identical.

In short, DVT screening should be given due importance in patients with gynecological malignant tumor, especially for those with old age, hypertension, hyperlipemia, adenocarcinoma, or history of radiotherapy, and rehabilitation exercises should be encouraged in these patients.

### Limitations of the study

First, many factors are associated with the occurrence of DVT, but the current study focused only on some factors, and the other factors including perioperative bleeding, occupation, infection, rheumatological disease, and nephrotic syndrome may also affect the occurrence of DVT adversely,^11^ but they were not included and studied. Secondly, in the current study, because most of the included patients were treated surgically, it was difficult to determine the influence of surgical treatment on the occurrence of DVT. Hence, more studies, especially some multi-center clinical studies on large scales should be carried out in the future.

### Authors’ Contribution

**QL and LL:** Conceived, designed and did statistical analysis & editing of manuscript.

**QL, YLX, YP and LL:** Did data collection and manuscript writing, and final approval of manuscript.
